# Delayed Corneal Endothelial Cell Loss Following CyPass Stent Insertion: A Case Report

**DOI:** 10.7759/cureus.109265

**Published:** 2026-05-20

**Authors:** Catherine M Ouyang, Alexander G Southall, Keith S Ong

**Affiliations:** 1 Ophthalmology, Sydney Eye Hospital, Sydney, AUS; 2 Engineering, The University of Sydney, Sydney, AUS; 3 Ophthalmology, Royal North Shore Hospital, Sydney, AUS

**Keywords:** corneal endothelial cell loss, cytotoxicity, foreign body reaction, minimally invasive glaucoma surgery, surgical glaucoma

## Abstract

We report a case of delayed corneal endothelial cell loss (CECL) following CyPass® stenting in a 71-year-old female with normal-tension glaucoma. CECL is a known complication of the CyPass® stent, which resulted in its withdrawal from the market in 2018; however, the mechanism for CECL is often attributed to mechanical endothelial disruption from poorly positioned stents. In this case, our patient suffered around a 40% CECL, which first started five years following stent implantation and responded well to ripasudil therapy. This occurred despite it being optimally positioned, prompting an alternate explanation for the mechanism of CECL following CyPass® stent insertion. We propose three areas for future research, which may explain the CECL: mechanical stress, such as from device-cornea touching; inflammation, from a chronic foreign body reaction; and direct cytotoxicity from degradation of irradiated polyimide material. Further research is needed to establish the biocompatibility of irradiated polyimide in the eye, as well as the role of ripasudil in stabilizing minimally invasive glaucoma surgery-related CECL.

## Introduction

In recent years, minimally invasive glaucoma surgery (MIGS) has grown in popularity as a bridge between topical therapies and traditional glaucoma surgeries [1]. MIGS involves the placement of a small device into the anterior chamber of the eye with the aim of increasing the drainage of aqueous flow. There are two main ways by which MIGS can achieve this effect: either by promoting outflow through the trabecular meshwork and into the canal of Schlemm (e.g., devices such as iStent and Hydrus Microstent), or through increasing the aqueous flow through alternate pathways such as the uveoscleral pathway (e.g., devices such as the CyPass® stent). Compared with traditional surgeries such as trabeculectomies, MIGS is considered to be a safer procedure but achieves more modest intraocular pressure (IOP)-lowering effects.

The CyPass® stent (Alcon, USA) is a form of MIGS placed in the supraciliary space. Introduced to the market in 2016, the CyPass® stent exerts its IOP-lowering mechanism uniquely by increasing the outflow of aqueous humour through the uveoscleral pathway directly into the suprachoroidal space. The stent was later withdrawn from the market in 2018, due to a high rate of delayed corneal endothelial cell loss (CECL) [2]. As corneal endothelial cells are vital in maintaining corneal transparency and regulating fluid and ion transport into the stroma of the cornea, declining corneal endothelial cell density (CECD) can result in corneal edema, bullous keratopathy, perforation, and permanent visual impairment [3]. As the rates of CECL were highest among patients with malpositioned or protruding shunts, the mechanism for CECL was attributed to mechanical trauma from intermittent device-to-cornea touching [2]. However, this explanation may not account for the CECL noted in patients with well-positioned stents; in the COMPASS-XT trials, the rate of patients with significant CECL (>30% of baseline) with well-positioned stents (no or one retention ring showing) was still double that of the control, phacoemulsification-only group [2]. These findings generated ambiguity regarding the etiology and ongoing management of patients with CyPass® stents in situ, particularly considerations surrounding stent explantation when stents were well-positioned.

This case explores other possible mechanisms for delayed CECL outside of mechanical malposition, which represent areas for further investigation. Alternative mechanisms to explain CECL have been proposed in the literature, including reports of foreign body reactions to the CyPass® stent [4]. We also speculate that the polyimide stent can cause corneal endothelial cytotoxicity resulting from delayed material breakdown.

## Case presentation

A 71-year-old female of East Asian descent with normal-tension glaucoma and high myopia underwent sequential phacoemulsification in June 2018 and trabeculectomy in December 2018 in the right eye, and subsequent combined phacoemulsification with insertion of a CyPass® stent in the left eye in August 2018. She had previously been treated for her glaucoma with topical therapy as well as selective laser trabeculoplasty in both eyes. The highest IOP recorded preoperatively in either eye was 18 mmHg during a water drinking test (Table 1). Her other ocular history included a 2 mm nasal corneal scar from resolved contact-lens-associated keratitis in the left eye.

**Table 1:** Comparison of preoperative and postoperative features. *: Serial measurements from 2017 to 2018. ^†^: Serial measurements from 2019 to 2025.

	Preoperative, June 2018		Postoperative, August 2024	
	Right	Left	Right	Left
Best corrected visual acuity (m)	6/9	6/9	6/9	6/9
Intraocular pressure (mmHg)	12–18^*^	12–18^*^	10–14^†^	12–16^†^
Central corneal thickness (µ), Tomey EM-4000 (Nürnberg, Germany)	543	524	526	524
Anterior chamber depth (mm), Zeiss IOLMaster 700 (California, USA)	3.11	3.15	4.69	4.44

**Figure 1:**
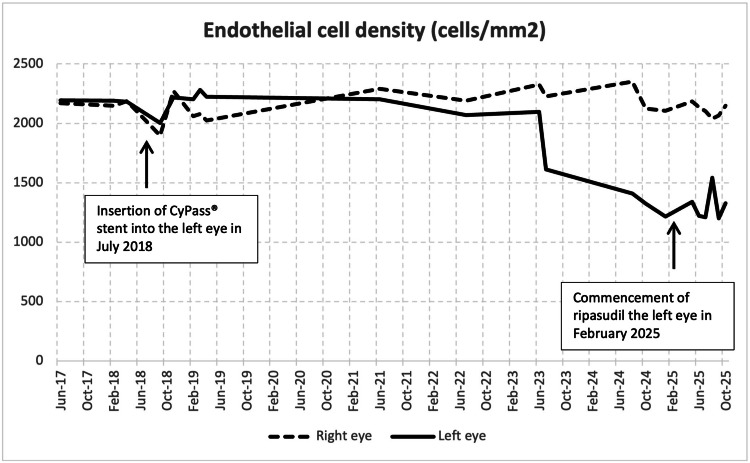
Comparison of endothelial cell densities from 2017 to 2025. The CyPass stent was inserted in the left eye in July 2018. The right eye underwent phacoemulsification and insertion of an intraocular lens in June 2018, followed by trabeculectomy in December 2018. The left eye underwent combined phacoemulsification and CyPass stenting in July 2018, and ripasudil commenced in February 2025. Note the marked onset of left-sided endothelial cell loss in June 2023, five years after CyPass stent insertion.

**Figure 2:**
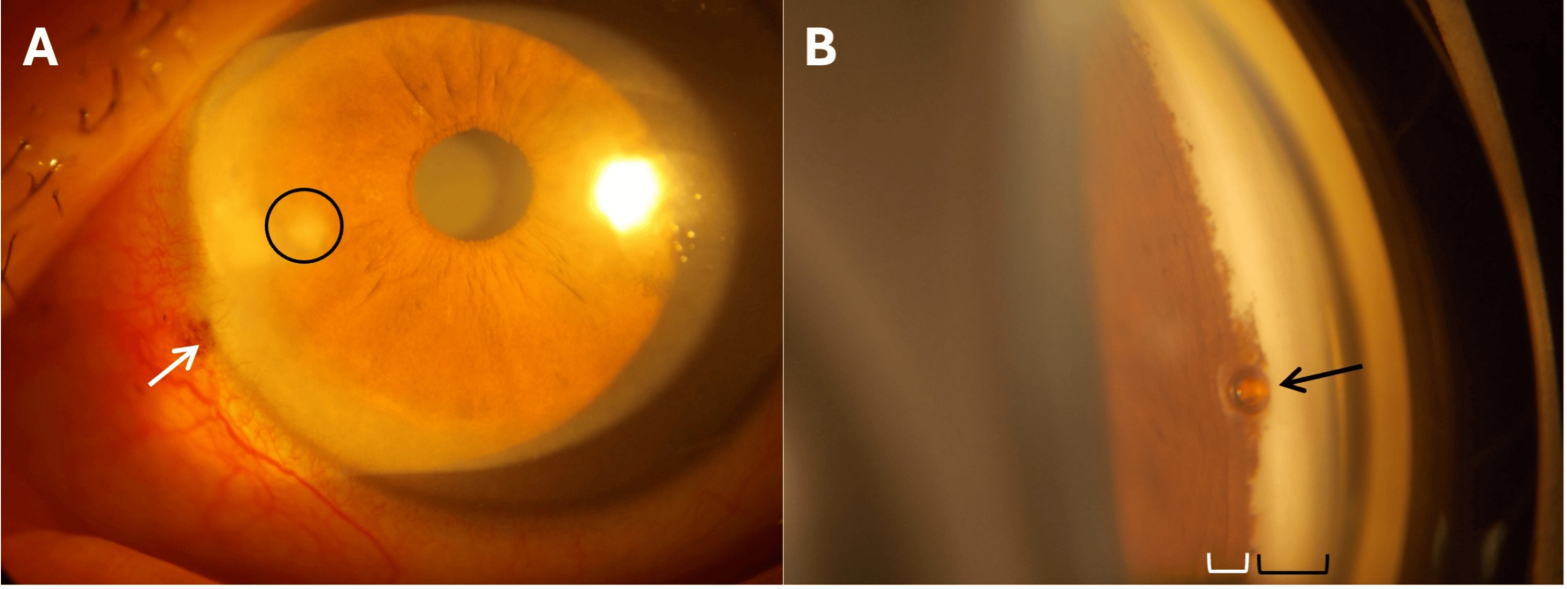
Position of the CyPass® stent in the left eye. A: Slit lamp image. The CyPass® stent is situated at the 8 o’clock position and is not visible (white arrow). The cornea is clear and without edema. A nasal scar from previous contact-lens keratitis can be noted (black circle). B: Gonioscopic view of the CyPass® stent (black arrow) in the supraciliary space between the ciliary body (white line) and scleral spur (black line) of the left eye. Note the absence of any retention rings showing, indicating minimal stent protrusion and potential for contact with the cornea.

A significant decline in the CECL of her CyPass® stented left eye was first noted in July 2023 (five years postoperatively), while her right, trabeculectomy-treated eye had stable CECL levels over the seven-year follow-up period (Figure 1). This was identified via monitoring of serial specular microscopy (Tomey® EM-4000, Nürnberg, Germany) results, and the patient did not complain of any visual disturbance. By October 2025, the CECD in the left eye had decreased by 40% to a final count of 1,327 cells/mm² (from 2,181 cells/mm²) (Table 2). Specular microscopy demonstrated generalized CECL without any preference for the region surrounding the CyPass® stent. Gonioscopy confirmed that her stent was appropriately positioned in the anterior chamber angle at 8 o’clock with no retention rings showing (Figure 2). Her anterior chamber angle was wide open in all quadrants. There were no clinical features of epithelial or stromal corneal edema, sectoral differences of the corneal endothelium, or any signs of chronic intraocular inflammation. There were no features suggestive of an alternate cause of CECL, such as corneal dystrophy.

**Table 2:** Timeline of change in corneal endothelial cell density and coefficient of variation preoperatively and postoperatively. Coefficient of variation data is not available between June 2017 and April 2018. *: Measurement taken two weeks before cataract surgery in the right eye. ^†^: Measurement taken one month before trabeculectomy in the right eye. ^‡^: Measurement taken one month before combined cataract surgery and insertion of CyPass® stent in the left eye. ˆ: measurement taken one month before the commencement of ripasudil. ^a^: Measurements were an average of three readings. ^b^: Measurements were an average of two readings.

	Corneal endothelial cell density (cells/mm^2^)		Coefficient of variation (%)	
	Right eye (trabeculectomy)	Left eye (CyPass® stent)	Right eye (trabeculectomy)	Left eye (CyPass® stent)
June 17	2,169	2,194	NA	NA
February 18	2,150	2,190	NA	NA
April 18	2,189	2,181	NA	NA
June 18	2,324*	1,994^‡^	39*	37^‡^
September 18	1,897	2,002	45	48
November 18	2,273^†^	2,218	38^†^	38
February 19	2,061	2,202	39	43
March 19	2,078	2,283	44	43
April 19	2,025	2,225	40	39
June 21	2,291	2,204	41	44
July 22	2,191	2,071	42	42
June 23	2,327	2,097	40	54
July 23	2,227	1,613	37	41
August 24	2,351	1,410	47	38
October 24	2,124	1,326	47	42
January 25	2,106	1,215ˆ	50	41
May 25^a^	2,186	1,341	42	41
June 25^a^	2,136	1,222	42	39
July 25^b^	2,103	1,210	47	42
August 25^a^	2,041	1,543	46	50
September 25^a^	2,068	1,201	44	37
October 25^a^	2,150	1,327	47	46

She otherwise had good IOP control in both eyes without any need for topical therapy over the course of her follow-up. Her corrected vision was stable at 6/9 in each eye. This correlated with stable visual fields and peripapillary retinal nerve fiber layer thickness. Central corneal thickness in the right and left eyes was 526 µ and 524 µ, respectively (Table 1).

She was diagnosed with Cypass® stent-associated CECL. She was commenced on ripasudil 0.4% four times daily to the left eye in February 2025 to preserve CECD, and her CECL subsequently plateaued (Table 2).

## Discussion

This case raises two questions: first, about the mechanism of CECL following CyPass® stenting, and second, regarding the management of CECL secondary to CyPass® stenting or MIGS.

There is emerging evidence to suggest that MIGS are associated with a greater amount of CECL than implant-free procedures such as trabeculectomy [1]. In particular, the delayed onset of CECL at four years post-insertion of the CyPass® stent indicates that CECL is more likely to arise out of cumulative exposure to the stent, over initial surgery-related trauma [2]. The proposed causes of MIGS-associated CECL may broadly be categorized into mechanical, inflammatory, and cytotoxic causes. Mechanical causes, particularly touching between the device and corneal endothelium from blinking, eye rubbing, or positional changes during sleep, are the most well-supported mechanism of CECL in CyPass®-stented eyes [2]. However, the minimal amount of stent protrusion, anterior chamber depth, and angle width on gonioscopy (Figure 2) makes it geometrically unlikely for intermittent device-corneal touching to be a significant feature of our case. A proportion of patients in the COMPASS-XT trial, as well as in this case, experienced significant CECL despite an optimally positioned stent without overt signs of device-corneal contact [2]. It is also possible that implanted materials may alter corneal biomechanics and aqueous humour drainage, though this effect has not been investigated with the CyPass® shunt, nor is it known how this may influence CECD. In our case, there were no regional differences in the amount of CECL to support a mechanical etiology.

There is evidence that supports a chronic inflammatory contribution to CyPass®-associated CECL. Several other case reports have described foreign body reactions found in explanted CyPass® stents [4-7] or to other polyimide implants in vivo [8,9]. Habbe et al. previously described a series of 14 explanted stents for CECL, wherein histopathology confirmed fibrous obliteration and encapsulation of the stent and chronic foreign body-related granulomatous inflammation [4]. All cases retained adequate IOP control when the stent was in situ. Schoelles et al. reported a case of foreign body granuloma noted in an explanted stent, which presented with chronic intraocular inflammation and recurrent IOP rises [5]. Another case series published by Hübner et al. described fibrotic tissue reactions in two-thirds of explanted stents [6]. Indications for explantation in the report included elevated IOP, corneal decompensation, and persistent hypotony [6]. Interestingly, in our case, no signs of active or previous inflammation were noted within the eye.

We propose that there is a direct cytotoxic effect of the CyPass® stent, arising from material, manufacturing, or sterilizing variances, which may alter the biocompatibility of the polyimide material. Polyimides represent a group of structurally diverse polymers that are widely considered to be biocompatible and inert. However, variations in its molecular architecture may alter its compatibility and cytotoxicity [10]. The majority of research demonstrating its safety has predominantly focused on human vascular endothelial cells in vitro [11] or as a neural implant within the cerebral cortex or retina in vivo, where no intraocular inflammation was noted [12]. The CyPass® stent was evaluated during pre-clinical stages with implantation into the suprachoroidal space in rabbits, and demonstrated no signs of an inflammatory reaction within the 180-day test period [13]. However, the biostability of the polyimide stent material within the aqueous environment over an extended duration remains to be evaluated.

The sterilization process for the CyPass® stent may also have the potential to influence its biocompatibility; however, further research in this area is required to establish whether there is any effect on cytotoxicity and corneal endothelial health. The stent is sterilized using electron beam irradiation, which has recently been found to cause chemical and structural changes in polyimides, including in the formation of free radicals and the cleavage of polymer chains [14,15]. Despite evidence demonstrating the biocompatibility of polyimides, these studies may not be valid when considering the biocompatibility of irradiated polyimides. Biocompatibility testing with consideration to byproducts of the manufacturing and sterilization process may have a useful role in minimizing adverse device-related reactions.

Sporadic case reports have also emerged, which propose that polyimide materials have the potential to improve stability over time. In other ophthalmic settings, polyimide has been used for the haptics of intraocular lenses. Case reports of explanted lenses due to haptic breakage have noted an unusual loss of elasticity and brittle transformation of the polyimide material, though no foreign body reactions were noted [16-19]. One case was associated with pseudophakic bullous keratopathy [16], though no CECL was described in other cases. This may further imply that the biomechanical properties of polyimide can change over time, and potentially correspond with changes in its biostability.

These cases raise the possibility for other explanations of CECL, other than from the mechanical theory of endothelial cell loss. Other possible mechanisms to further explore in future research include the presence of a chronic foreign body inflammatory process from the stent to cause CECL, and whether there are degradation or radio-degradation products of the stent, which may influence endothelial cell viability.

Our case also highlights issues facing the recognition and management of MIGS-associated CECL, including possible medical and surgical approaches. Early stages of CECL may be asymptomatic, as occurred in our patient, and monitoring of her CECD was only performed as CECL was a known complication of the CyPass® stent. As CECD continues to decline, patients may note reduced visual acuity and glare as a result of corneal edema and increasing stromal thickness [3]. In the late stages of CECL, corneal edema may coalesce into bullae and necessitate corneal transplantation [3]. Given the devastating consequences of CECL if identified in the late stages, regular monitoring and early detection of changes in CECD may be beneficial in patients with CyPass® stents.

Stent removal in our patient was considered in January 2025; however, following the plateau of CECL with the commencement of ripasudil, an ongoing conservative approach with CECD monitoring may be a better option. Ripasudil is a rho kinase inhibitor, which has demonstrated an endothelial protective effect in patients with CECL post-cataract surgery [20]. Although our patient demonstrated a favorable response, more research is required to evaluate its efficacy for use in MIGS-associated CECL.

Removal of the offending stent offers a permanent solution; however, the risks associated with this procedure were considered to be high. As can be seen in Figure 2, there was a minimally exposed length of stent that could be grasped, making it surgically difficult to remove the stent. Moreover, the patient would require a subsequent glaucoma operation or re-commencement of topical therapy to adequately manage their IOP.

Limitations

As a single case study, this report features several key limitations. The nature of the alternative proposed mechanisms for CECL remains highly speculative and theoretical. The relationship between irradiation and polyimide biocompatibility, as well as the presence of foreign body reactions, represents areas that require further in vivo research to establish whether any relationship is present. In addition, the lack of any chronic inflammatory reaction to be observed in our patient makes it less likely for this to be a significant feature of our case. Although the geometry of the CyPass® stent in the anterior chamber angle minimizes the likelihood that device-cornea touch is a key contributor to CECL, no dynamic imaging was performed to definitively exclude this potential. Lastly, while ripasudil may have a corneal endothelial protective effect, larger-scale observational or randomized controlled trials are required to determine whether it has a role in managing MIGS-associated CECL.

## Conclusions

This case describes the history and management of a patient with severe CECL following CyPass® stenting, despite appropriate stent positioning. While previous theories have attributed CyPass®-associated CECL to mechanical endothelial damage, this report raises the possibility of alternative mechanisms for CECL, including foreign body reactions or polyimide-associated endothelial toxicity. There may also be a benefit for the use of rho kinase inhibitors, such as ripasudil, to halt the progression of CECL following MIGS. Further research is required to establish the biocompatibility of polyimide in the anterior segment of the eye and the use of ripasudil for MIGS-associated CECL.
